# Analysis of the community composition and bacterial diversity of the rhizosphere microbiome across different plant taxa

**DOI:** 10.1002/mbo3.762

**Published:** 2018-11-22

**Authors:** Shaonan Lei, Xiaohong Xu, Zhiqiang Cheng, Juan Xiong, Rongqin Ma, Lanlan Zhang, Xiaorong Yang, Yunxi Zhu, Binghuo Zhang, Baoyu Tian

**Affiliations:** ^1^ Provincial University Key Laboratory of Cellular Stress Response and Metabolic Regulation, College of Life Sciences Fujian Normal University Fuzhou China; ^2^ Engineering Research Center of Industrial Microbiology of Ministry of Education Fujian Normal University Fuzhou China; ^3^ Library Fujian Normal University Fuzhou China; ^4^ Jiujiang University Jiujiang China

**Keywords:** bacterial diversity, community composition, core microbiome, plant taxa, rhizobacteria

## Abstract

Rhizobacteria play an important role in bridging the soil and plant microbiomes and improving the health and growth of plants. In this study, the bacterial community structures and compositions of rhizosphere microbiomes associated with six plant species, representing two orders and three families of wild plants grown in the same field, were evaluated. The six plant species examined harbored a core and similar bacterial communities of the rhizosphere microbiome, which was dominated by members of Rhizobiales, Sphingomonadales, Burkholderiales, and Xanthomonadales of Proteobacteria, Subgroup 4 of Acidobacteria, and Sphingobacteriales of Bacteroidetes. Plant species had a significant effect on the microbial composition and Operational Taxonomic Unit (OTU) abundance of the rhizosphere microbiome. Statistical analysis indicated a significant differential OTU richness (Chao1, *p* < 0.05) and bacterial diversity (Shannon index, *p* < 0.0001) of the rhizosphere microbiome at the plant species, genus, or families levels. The paralleled samples from the same plant species in the PCoA and hierarchical cluster analysis demonstrated a clear tendency to group together, although the samples were not strictly separated according to their taxonomic divergence at the family or order level. The CAP analysis revealed a great proportion (44.85%) of the variations on bacterial communities could be attributed to the plant species. The results demonstrated that largely conserved and taxonomically narrow bacterial communities of the rhizosphere microbiome existed around the plant root. The bacterial communities and diversity of the rhizosphere microbiome were significantly related to the plant taxa, at least at the species levels.

## 1 INTRODUCTION

The microbes in the rhizosphere are a diverse mixture of microorganisms that can actively interact with the host plant in different ways. Since the rhizosphere represents the interface between the soil and plants, the rhizosphere microbiome is thought to have substantial importance in bridging the soil and plant microbiomes and improving plant host health and soil fertility (Berg et al., 2005; Bulgarelli, Schlaeppi, Spaepen, Themaat, & Schulze‐Lefert, 2013; Turner, James, & Poole, 2013). The rhizobacterial microbiota improves plant health by protecting the plant hosts from phytopathogens, providing them with relevant nutrients by biologically fixing nitrogen, and producing phytohormones to promote plant growth or enhance plant fitness (Berendsen, Pieterse, & Bakker, 2012; Bulgarelli et al., 2013; Mendes, Garbeva, & Raaijmakers, 2013; Pii et al., 2015; Spaink, 2000; Tian, Yang, & Zhang, 2007). To fully understand the functions and activities of the rhizosphere microbiome for the beneficial management of plant health, it is necessary to explore the composition, assembly, and variation of the microbial communities that are present in the rhizosphere and the underlying mechanisms that drive microbiome assembly.

Recently, the communities, composition, and variation of the plant root‐associated microbiome from several plant species, such as the model plant species *Arabidopsis* (Bulgarelli et al., 2012; Lundberg et al., 2012; Schlaeppi et al., 2014), and economically important crop plants such as maize (Peiffer et al., 2013), rice (Edwards et al., 2015; Knief et al., 2011), potato (Rasch et al., 2006), tomato (Tian, Cao, & Zhang, 2015), tobacco (Robin et al., 2006), and soybean (Mendes, Kuramae, Navarrete, Veen, & Tsai, 2014; Xu et al., 2009), have been revealed using culture‐independent 16S rRNA gene‐based sequencing techniques. These studies have given us a glance about the bacterial community, composition, and diversity of the rhizosphere microbiome and its relationship with the soil microbiome. The community structure and composition of the plant‐associated microbiome depends on several factors, such as the soil properties, plant nutritional status, climate, plant genotype, and even the developmental stage of the host plant (Bulgarelli et al., 2015; Pii et al., 2016; Trognitz, Hackl, Widhalm, & Sessitsch, 2016; Turner, James, et al., 2013). Plants recruit their own microorganisms from the surrounding soil and provide entry into the root. Soils provide the bacterial inoculum and serve as a pool of bacterial species present in each soil type (Bulgarelli et al., 2013; Dombrowski et al., 2017; Pii et al., 2016). Therefore, it is clear that both soil type and plant species affect the microbial community and composition of the rhizosphere microbiome (Berg & Smalla, 2009; Bulgarelli et al., 2012; Inceoglu, Abu Al‐Soud, Salles, Semenov, & Elsas, 2011; Lundberg et al., 2012). However, the effects of the factors on the community compositions in the rhizosphere and endosphere microbiomes were significantly different. For instance, plant hosts, including plant species, genotype, and also the plant developmental stage, are a strong determinant of the endophytic bacterial community (Aleklett, Leff, Fierer, & Hart, 2015; Bulgarelli et al., 2012; Fitzpatrick et al., 2018; Inceoglu et al., 2011; Lundberg et al., 2012; Miyambo, Makhalanyane, Cowan, & Valverde, 2016; Naylor, Degraaf, Purdom, & Coleman‐Derr, 2017;Schlaeppi et al., 2014).

Compared with the bulk soil and endosphere environment, the biomass and activity of microorganisms in the rhizosphere are enhanced as a result of the exudation of compounds by the roots (Chaparro et al., 2013; Raaijmakers, Paulitz, Steinberg, Alabouvette, & Moënne‐Loccoz, 2009; Stringlis et al., 2018). Therefore, plants can also influence the structure and function of the bacterial communities in the rhizosphere soil around their roots. The studies on the microbial rhizosphere communities have shown the significant influence of plant species and cultivars in shaping microbial communities in the rhizosphere, including studies on different cultivars of potato (Inceoglu et al., 2011; Weinert et al., 2011), maize (Peiffer et al., 2013), Arabidopsis (Bulgarelli et al., 2012; Lundberg et al., 2012), rice (Edwards et al., 2015; Knief et al., 2011), and soybean (Mendes et al., 2014; Xu et al., 2009), or intra‐species comparison (Bouffaud, Poirier, Mulle, & Moënne‐Loccoz, 2014; Bulgarelli et al., 2015; Ofek, Voronov‐Goldman, Hadar, & Minz, 2014; Pongsilp, Nimnoi, & Lumyong, 2012; Schlaeppi et al., 2014; Turner, Ramakrishnan, et al., 2013; Wieland, Neumann, & Backhaus, 2001). Even different genotypes of the same plant species have also an effect on the bacterial community structure and composition of their rhizosphere microbiome (Marques et al., 2014; Rasch et al., 2006; Robin et al., 2006). These studies have demonstrated that the diversification in the community structure of the rhizosphere microbiome can be partially explained by the phylogenetic distance of the plant hosts. For example, Bulgarelli et al. (2015) found that the host genotype accounts for approximately 5.7% of the variance in the rhizosphere microbiome composition. However, the degree to which the plants contribute to the rhizobacterial communities and the underlying mechanisms by which the plants drive the rhizosphere microbiome are not well understood. The effects on the rhizosphere microbiome of plant species, cultivars, or even genotypes should be subjected to more research, but a study from the higher phylogenetic distance of plant hosts is lacking.

To examine the degree to which the plant taxa drive the assembly of bacterial communities in specific soil environments, we evaluated the bacterial community structures and compositions of rhizosphere microbiomes associated with six plant species representing two orders, three families, and six genera of wild plants grown in the same field using high‐throughput DNA sequencing techniques. Understanding the mechanisms that shape and drive the microbiome assembly in the rhizosphere will provide a basis on which to construct a healthy plant rhizosphere microbiome to benefit plant breeding, improve soil management strategies, and introduce universal biological control agents and fertilizers to develop more sustainable agricultural practices.

## 2 EXPERIMENTAL PROCEDURES

### 2.1 Soil collection and metagenomic DNA preparation

Six different plant species, including *Artemisia argyi*,* Ageratum conyzoides*,* Erigeron annuus*, and *Bidens biternata* of the Asterales order and *Euphorbia hirta* and* Viola japonica* of the Malpighiales order, were collected from a naturally developed lawn adjacent to a tomato experimental field on November 25, 2015, on the Qishan campus of the Fujian Normal University in Fuzhou, China (26°15′00″N, 119°12′00″E; Supporting Information Table S1). Among these plants, *V*. *japonica* is a perennial herb plant, and the other five plants are annual or biennial (*E*. *annuus*) herb plants. Plants in the late vegetative stage (6 or 7 months old) were harvested separately, and the roots were shaken to remove the large soil particles. The soil that attached tightly to the roots was carefully collected with a sterile filter paper strip and used as the source of rhizosphere soil (Bulgarelli et al., 2012; Tian et al., 2015). For each plant, five replicates were randomly collected. Therefore, a total of 30 rhizosphere soil samples were obtained (Supporting Information Table S1).

The total genomic DNA was separately extracted using a Power Soil^®^ DNA Isolation Kit (Mo Bio Laboratories, Carlsbad, CA, USA) according to the manufacturer's instructions. The extracted genomic DNA was dissolved in 50 μL of elution buffer and stored at −20°C for subsequent sequencing.

### 2.2 PCR amplification and high‐throughput sequencing

The concentration and purity of the metagenomic DNA extracted were measured using a spectrophotometer (NanoDrop 2000, Thermo Scientific, Waltham, MA, USA). Approximately, 400 bp DNA fragments of the bacterial 16S rRNA gene targeting the hypervariable region V3–V4 were amplified using the primer pair 341F (5′‐CCTACGGGNGGCWGCAG‐3′) and 805R (5′‐GACTACHVGGGTATCTAATCC‐3′) fused with the Illumina MiSeq adaptors and a 6 bp barcode sequence unique to each sample (Tian et al., 2015). The PCR amplification products were subsequently purified, combined in equimolar ratios, and subjected to high‐throughput sequencing on an Illumina MiSeq sequencing platform to produce paired 250‐nucleotide reads at Sangon Biotech (Shanghai, China).

### 2.3 Data processing and bacterial community analysis

The raw sequence was spliced using FLASH (version 1.2.3), which can generate much longer reads by overlapping and merging read pairs (2 × 250 bp) before assembling a gene segment (Magoč & Salzberg, 2011), and adaptors, barcodes, and primers were removed using Cutadapt (version 1.9.1). Sequences with ambiguous bases, average quality scores <25, or lengths shorter than 200 bp were removed to control sequence quality. Chimeric sequences were identified and removed with a de novo method using USEARCH (version 8.1.1861) (Edgar, 2010). After the removal of the chimera, high‐quality bacterial sequences were collected for subsequent analysis. A summary of data processing steps is provided in Supporting Information Table S2.

To correct for the differences in sequencing depth, bacterial read numbers per sample were rarefied to the smallest number of reads. Effective bacterial sequences were separately subsampled for each sample for the subsequent statistical analysis. After subsampling, the data were processed using a modified SOP pipeline based on USEARCH and the software package QIIME (Caporaso et al., 2010; Tian et al., 2015). Briefly, the selected sequences were clustered to Operational Taxonomic Units (OTUs) using a two‐stage clustering algorithm with USEARCH (version 8.1.1861) at 97% sequence identity (Edgar, 2010). Representative sequences in each OTU were aligned to the SILVA reference alignment (Database release 128 updated September 2016) (Yilmaz et al., 2014). Taxonomy was subsequently assigned to each representative sequence using RDP with a minimum confidence of 85%.

### 2.4 Statistical analyses

The diversity index and species richness estimator (α‐diversity) for each sample, including OTU richness, Chao‐1 diversity, ACE diversity, and the Shannon index, with respect to a sequence depth of 3%, were calculated using QIIME script function *alpha_diversity.py* (version 1.8.0; Supporting Information Table S3). Rarefaction and rank‐abundance curves were calculated at a level of 97% similarity of the OTUs. Statistical analysis was performed using an analysis of variance (ANOVA) with *p* values to determine whether the diversity indices or species richness estimators were statistically significantly different among the plant rhizosphere soil samples (Cúcio, Engelen, Costa, & Muyzer, 2016). In addition, the statistically significant differential OTUs (*p* < 0.05) in the different sample groups were identified on a normalized OTU table by comparing OTU frequencies of the within‐group to the between‐group using the QIIME script function *group_significance.py* (Caporaso et al., 2010). Relative abundances of the 100 most differentially abundant OTUs in each sample were visualized by drawing a heatmap. To estimate the beta diversity, weighted UniFrac distances were used to calculate the similarities of the memberships and structures found in the various plant species at the OTU levels (QIIME script function *beta_diversity.py*). PCoA plots were used to visualize the difference in bacterial community and compositions of the plant‐associated microbiome. Canonical Analysis of Principle Coordinates (CAP) was computed using the function capscale from the R Package Vegan (Anderson & Willis, 2003; Oksanen et al., 2015). Variance partitioning and significances on bacterial communities for experimental factors, including the taxonomy, life_cycle, and root_system, were determined by running a permutation‐based ANOVA test using 999 permutations (Supporting Information Table S2).

## 3 RESULTS

### 3.1 Sequencing quality control and summary

A series of processes were used to control sequence quality: screening, filtering, preclustering processes, and chimera removal, resulting in 822,483 reads of high‐quality bacterial 16S rRNA V3‐V4 gene sequences; an average of 27,416 ± 2,228 reads per sample (min = 20,850, max = 32,016) was obtained (Supporting Information Table S3). The bacterial read numbers per sample were rarefied to the smallest number of reads. In this case, 20,850 effective bacterial sequences were randomly extracted for subsequent statistical analysis (Supporting Information Table S4).

### 3.2 Community structures and compositions of the rhizosphere microbiomes associated with six plant species

**Figure 1:**
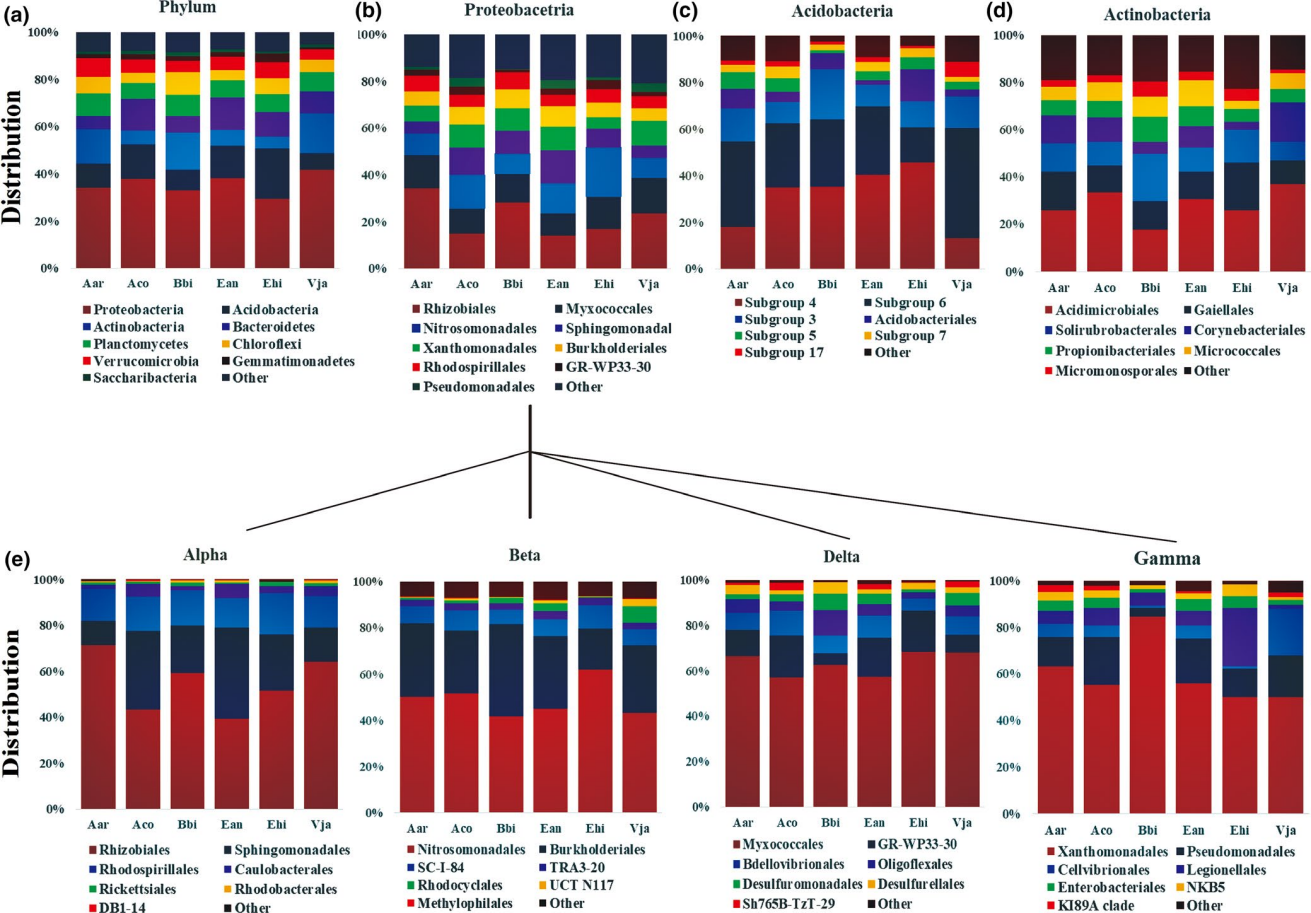
The composition and relative abundance of major bacterial taxa in rhizosphere soil of six plant species. Each bar represents the average value of five replicates in each sample group. (a) The composition and relative abundance of major bacterial phyla; (b–d). The composition and relative abundance of major bacterial orders from the phyla of Proteobacteria (b), Acidobacteria (c), and Actinobacteria (d). (e). The composition and relative abundance of major bacterial orders from four classes of the phylum Proteobacteria: Alpha‐proteobacteria, Beta‐proteobacteria, Delta‐proteobacteria, and Gamma‐proteobacteria. Aco, *Ageratum conyzoides*; Ean, *Erigeron annuus*; Bbi, *Bidens biternata*; Aar, *Artemisia argyi*; Vja, *Viola japonica*; Ehi, *Euphorbia hirta*

The randomly extracted sequences were clustered to OTUs with an average of 2,819 ± 273 OTUs per sample (min = 2,272, max = 3,275; Supporting Information Table S4). Representative sequences in each OTU were compared with the SILVA database to assign a taxonomy classification to determine the community structures and compositions of the plant rhizosphere microbiomes (Figure 1 and Supporting Information Figure S1). The rhizosphere microbiome in the six plant species was dominated by members of Proteobacteria (35.66% ± 3.99%), followed by Acidobacteria (12.63% ± 4.67%), Actinobacteria (10.77% ± 4.91%), Bacteroidetes (9.93% ± 2.95%), Planctomycetes (8.05% ± 0.96%), Chloroflexi (6.18% ± 1.86%), Verrucomicrobia (5.86% ± 1.23%) among others (Figure 1a). Rhizobiales (7.78% ± 2.58%) and Sphingomonadales (3.23% ± 1.29%) of Alpha‐proteobacteria (13.91% ± 2.09%), Nitrosomonadales (4.28% ± 1.24%) and Burkholderiales (2.50% ± 0.54%) of Beta‐proteobacteria (8.66% ± 1.66%), Myxococcales (4.53% ± 0.91%) of Delta‐proteobacteria (7.13% ± 1.04%), and Xanthomonadales (3.15% ± 0.99%) of Gamma‐proteobacteria (5.45% ± 1.96%) were highly abundant in Proteobacteria (Figure 1e), similar to that of Subgroup 4 (4.37% ± 2.88%) and Subgroup 6 (3.50% ± 0.53%) in Acidobacteria, Acidimicrobiales (3.01% ± 1.61%) in Actinobacteria and with Sphingobacteriales (7.35% ± 2.33%) and Cytophagales (1.73% ± 0.79%) in Bacteroidetes (Figure 1 and Supporting Information Figure S1). Few archaeal OTUs were detected in the rhizosphere soil samples of the six plant species.

Core microbiome analysis using QIIME software covering all six plant species revealed a total of 1,109 core OTUs belonging to 113 bacterial genera of 25 classes, accounting for 73.46% of the total sequencing data. The predominant genera (above 1% of the total reads belonging to core OTUs) included *Blastocatella*,* Ferruginibacter*,* Bradyrhizobium*,* Variibacter*,* Sphingomonas*,* Variovorax*,* Acidibacter,* and some of unclassified bacteria. These predominant genera identified were composed of Rhizobiales, Rhodospirillales, Sphingomonadales, Burkholderiales, Nitrosomonadales, Myxococcales, and Xanthomonadales of Proteobacteria, Subgroup 4 and 6 of Acidobacteria, Acidimicrobiales of Actinobacteria, and Sphingobacteriales of Bacteroidetes (Supporting Information Table S5).

Variations in microbial community compositions and OTU abundance were observed in six different plant species For example, Proteobacteria was the predominant bacterial group of the rhizosphere microbiome, being the least represented (29.33% ± 2.50%) in *E. hirta* and the most represented (41.55% ± 2.59%) in *V. japonica* (Figure 1a). At a more detailed level, Rhizobiales (7.78% ± 2.58%) of Proteobacteria was highly enriched in *V. japonica* and *A. argyi*, and a higher proportion of Myxococcales (4.53% ± 0.91%) in *V. japonica*, similar to that of Nitrosomonadales (4.28% ± 1.24%) were observed in *E. hirta*, Sphingomonadales (3.23% ± 1.29%) and Burkholderiales (2.50% ± 0.54%) in *E. annuus*, Xanthomonadales (3.15% ± 0.99%) in *V. japonica*, demonstrating a highly varied community composition at the order level (Figure 1 and Supporting Information Figure S1). Similar results were also observed in other bacterial groups from the bacterial phyla Bacteroidetes and Acidobacteria. The OTUs belonging to Sphingobacteriales of Bacteroidetes showed a higher relative abundance in the samples of *A. conyzoides* and *E. annuus*. In contrast, the OTUs belonging to Acidobacteria Subgroup 4, Sphingobacteriales and Sphingomonadales were absent in *V. japonica* (Figure 1 and Supporting Information Figure S1).

**Figure 2:**
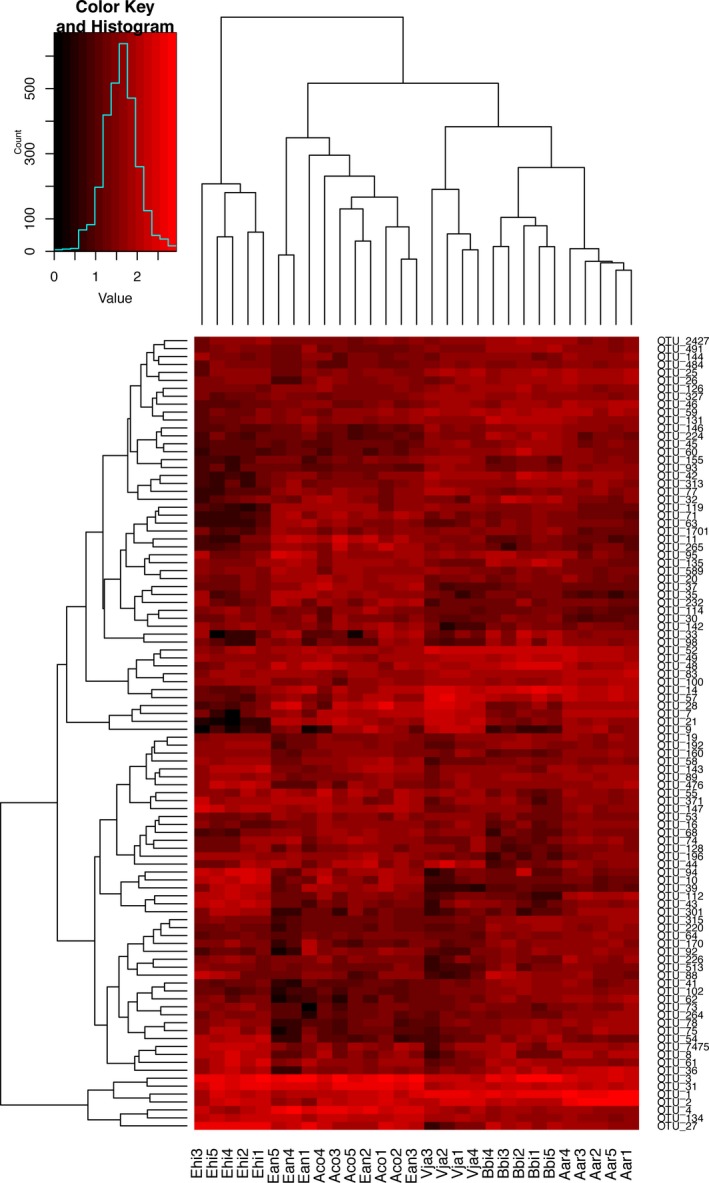
Heatmap depicting the relative abundance of the most 100 differentially abundant Operational Taxonomic Units (OTUs) in six plant rhizosphere soil sample. Dendrogram linkages and distances of OTUs are not phylogenetic, but based upon reads number (log transformed) of OTUs within the samples. Legend and scale shown in the upper right corner of the figure represent colors in heatmap associated with the relative abundance of OTUs (cluster of variables in *Y*‐axis) within each plant and soil sample (*X*‐axis clustering). Aco, *Ageratum conyzoides*; Ean, *Erigeron annuus*; Bbi, *Bidens biternata*; Aar, *Artemisia argyi*; Vja, *Viola japonica*; Ehi, *Euphorbia hirta*; Con, Bulk soils. The corresponding taxonomic profiles for each OTU were presented in Supporting Information Table S6

The analysis of variance (ANOVA) with *p* values (*p < *0.05) was used to identify statistically significant differential OTUs among the rhizosphere soil samples from six plant species. The reads from the identified differential OTUs, accounting for 74.51% of the total rarefied reads, primarily belonged to Sphingobacteriales of Bacteroidetes, Sphingomonadales, Rhizobiales, Nitrosomonadales, Xanthomonadales of Proteobacteria, and Subgroup 4 of Acidobacteria. The heatmap was constructed to visualize the variation of the relative abundance of the most 100 differentially abundant OTUs in the samples of six plants (Figure 2). Among these, the OTUs belonging to Rhizobiales, Sphingomonadales and Nitrosomonadales of Proteobacteria, Sphingobacteriales of Bacteroidetes, and Acidobacteria Subgroup 4 were significantly abundant in the most of the plant samples (Figure 2 and Supporting Information Table S6).

### 3.3 Estimating the bacterial diversity and species richness of the rhizosphere microbiomes in six plant species

Bacterial diversities in the samples of each plant (alpha diversity) were evaluated using an OTU‐based analysis method. Alpha diversities for all the samples are summarized in Supporting Information Table S4. The rarefaction curves showed that all the samples reached the saturation phase with a satisfactory level of confidence and a Good's coverage index of at least 94% (Supporting Information Table S4; Supporting Information Figure S2). Significant differential OTU richness estimated by Chao1 (*p* = 0.022 < 0.05) and bacterial diversity estimated by the Shannon index (*p* < 0.0001) were observed among the rhizosphere microbiome of six plant species. Among these, rhizobacteria of the plant *A*. *argyi* showed higher bacterial diversity (Shannon index: 10.069 ± 0.098) and OTU richness (Chao1: 4309.9 ± 144) compared with the rhizobacteria of the other five plant species. In contrast, *E. hirta* showed a lower bacterial diversity (Shannon index: 9.3542 ± 0.180) and OTU richness (Chao1: 3571.8 ± 120) than those of the other five plant species.

In addition, at the higher taxonomic levels, significant differential OTU richness estimated by Chao1 (*p* = 0.039 < 0.05) and bacterial diversity estimated by the Shannon index (*p* < 0.0001) were observed among the rhizosphere microbiome of the plant families of Euphorbiaceae, Asteraceae, and Violaceae. No significant differential OTU richness (Chao1: *p* = 0.229) and bacterial diversity (Shannon index: *p* = 0.144) were identified in the plant orders of Asterales and Malpighiales.

The rank‐abundance curve visually depicts both species richness and evenness in the six plant species. *Erigeron annuus*,* A*. *conyzoides*, and *A*. *argyi* exhibited higher species richness and evenness. In contrast, the *E*. *hirta*,* B*. *biternata,* and *V*. *japonica* samples showed lower species richness and evenness, suggesting that the bacterial species compositions of *E*. *annuus*,* A. conyzoides,* and *A*. *argyi* were more abundant and better distributed (Supporting Information Figure S3). Overall, the results demonstrated that plants grown in the same soil field had a significant effect on the bacterial diversity and species abundance of the microbial communities in the rhizosphere.

### 3.4 Estimating the dissimilarity and similarities of the rhizosphere microbiomes in six plant species using the beta diversity

**Figure 3:**
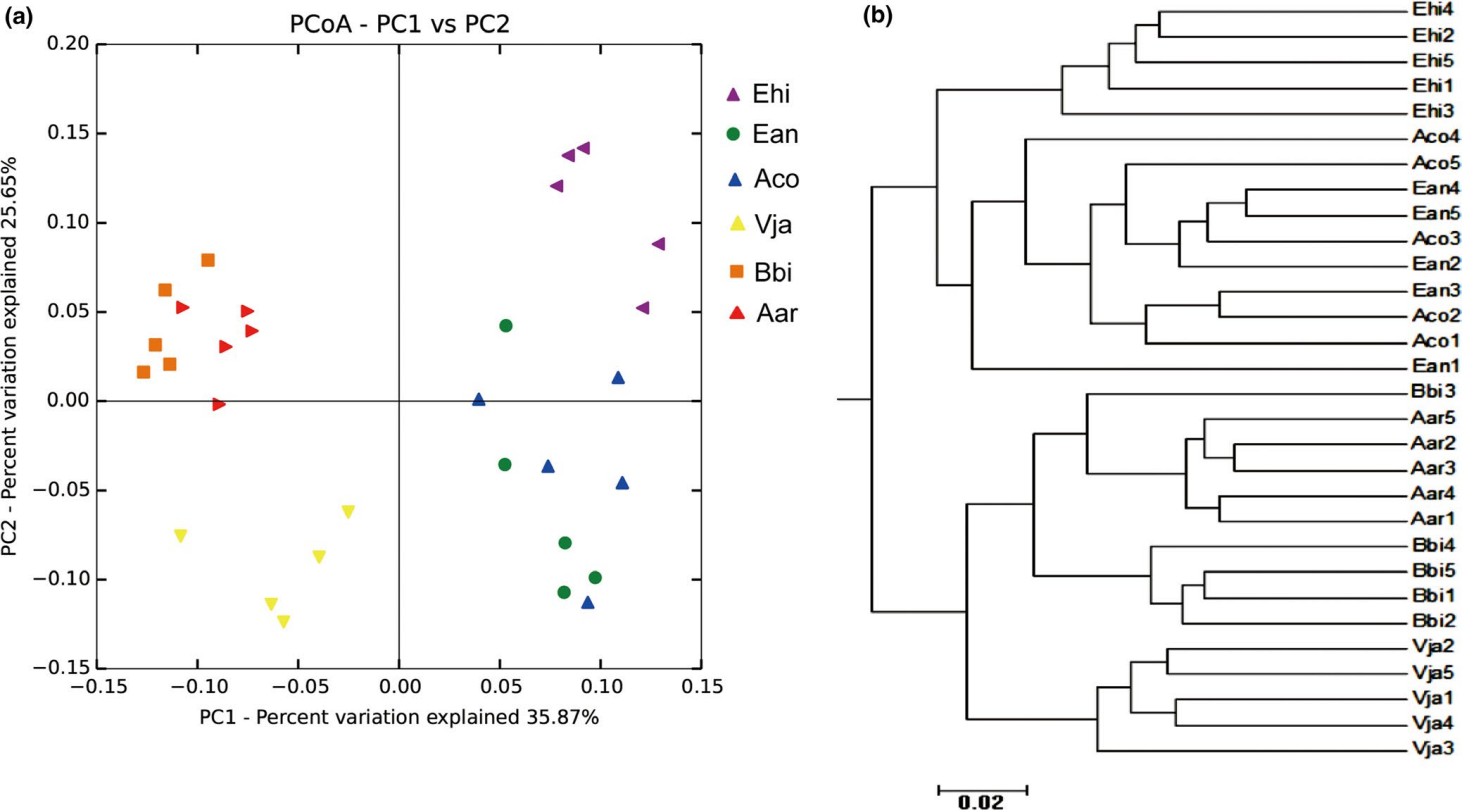
Beta diversity analysis to estimate the dissimilarity and similarity of bacterial communities and composition among different samples. (a) Principal coordinated analysis (PCoA) derived from dissimilarity matrix of weighted UniFrac distance. (b) The weighted UniFrac‐based cluster analysis of bacterial community composition among different samples. Aco, *Ageratum conyzoides*; Ean, *Erigeron annuus*; Bbi, *Bidens biternata*; Aar, *Artemisia argyi*; Vja, *Viola japonica*; Ehi, *Euphorbia hirta*

The principal coordinate analysis (PCoA), which assessed the beta diversity in the microbial structure and composition of the rhizosphere microbiome, revealed that the five paralleled samples from the same plant species demonstrated a clear tendency to group together. The data indicated distinctions in community structures and compositions in different plant species (Figure 3a). CAP analysis constrained to plant species, family, order, and the characteristics factors life_cycle and root_system revealed a prominent effect of plant species on bacterial communities (*p* = 0.001), explaining 44.85% of the variance (Supporting Information Figure S4). Similar results were also found in the UniFrac‐based hierarchical cluster analysis (Figure 3b). The results demonstrated that the samples significantly clustered into different groups based on their taxonomic divergence, although not for each plant species. The parallel samples of the plant species of *A*. *argyi*,* B. biternata,* and *V*. *japonica* obviously gathered as one group, separate from another group comprising samples from *E. hirta*,* A. conyzoides,* and *E. annuus* (Figure 3b). The two groups were further subgrouped, such as *V*. *japonica* from *A*. *argyi* and *B. biternata*, and *E. hirta* from *A. conyzoides* and *E. annuus*.

## 4 DISCUSSION

The rhizosphere microbiome, which is thought to have great importance to improve plant host health and productivity, has attracted more attention during the past several decades (Berg et al., 2005; Bulgarelli et al., 2013; Turner, James, et al., 2013). Previous studies have demonstrated the effect of plant species, cultivars, or genotypes on the rhizosphere microbiome (Bulgarelli et al., 2015, 2012 ; Lundberg et al., 2012; Marques et al., 2014; Peiffer et al., 2013). However, a few studies have been conducted to evaluate the effect of the plant hosts on the microbiome at intraspecies or higher taxonomic levels. The degree to which plants contribute to rhizosphere microbial communities is not well understood.

In this study, the bacterial community structures and compositions of rhizosphere microbiomes associated with a broad range of plant taxa were investigated. The rhizosphere microbiome in six plant species was primarily composed of Proteobacteria, Acidobacteria, Actinobacteria, and Bacteroidetes, with the most abundant bacterial groups from the bacterial orders of Rhizobiales, Sphingobacteriales, Myxococcales, Acidobacteria Subgroup 4, Nitrosomonadales, Acidobacteria Subgroup 6, Sphingomonadales, Xanthomonadales, Acidimicrobiales, and Burkholderiales. The investigation of the core microbiome revealed that six plant species shared a total of 73.46% of the rarefied reads that were primarily composed of the bacterial communities of Rhizobiales, Rhodospirillales, Sphingomonadales, Burkholderiales, Nitrosomonadales, Myxococcales, and Xanthomonadales of Proteobacteria, Subgroup 4 and 6 of Acidobacteria, Acidimicrobiales of Actinobacteria, and Sphingobacteriales of Bacteroidetes. The results demonstrated that plants grown in the same field harbored a similar microbial community and structure of the rhizosphere microbiome.

Plant species had a significant effect on the bacterial diversity and OTU abundance of the rhizosphere microbiome. The reads from the statistically significant differential OTUs (*p < *0.05) identified in the six plant rhizosphere samples accounted for 74.51% of the total sequenced data. The differential OTUs identified belonged to the dominant bacterial groups of the rhizosphere microbiome, including Sphingobacteriales, Sphingomonadales, Rhizobiales, Nitrosomonadales, Xanthomonadales, and Subgroup 4 of Acidobacteria. The differences of the rhizobacterial communities in different plant species resulted from the differential species richness and bacterial diversity of the rhizosphere microbiome. Statistical analysis indicated a strongly significant effect of plants from different species, genera, or families on the OTU richness (*p* < 0.05) and bacterial diversity (*p* < 0.0001) of the rhizosphere microbiome. The beta diversity analysis from PCoA and CAP agreed the results that a great proportion of the variations in microbial communities and composition across the rhizosphere soil samples could be attributed to the plant species, although the samples were not strictly separated according to their taxonomic divergence at the family or order level.

Proteobacteria, Bacteroidetes, Actinobacteria, and Acidobacteria comprised the predominant bacterial content of the six plant rhizosphere microbiomes. This result was largely consistent with previous investigations in other plants, such as tomato, maize, rice, and *Arabidopsis* (Bulgarelli et al., 2012; Edwards et al., 2015; Lundberg et al., 2012; Peiffer et al., 2013; Tian et al., 2015). The identification of the core microbiome in different plant species revealed the fact that the assembly of the communities and composition of the rhizosphere microbiome were driven by common selective forces. Alternatively, the bacterial community composition associated with the six plant species was also shown to be specific to their plant hosts. Plants from different species or families demonstrated a highly varied community composition at the level of the bacterial order, with highly enriched Rhizobiales, Myxococcales, and Xanthomonadales in *V. japonica*; Rhizobiales in *A. argyi*; Nitrosomonadales in *E. hirta*; and Sphingomonadales and Burkholderiales in *E. annuus*. Differing from previous observations with a minor fraction of the effects of plant cultivars or genotypes on the bacterial communities and the composition of the rhizosphere microbiome, a substantial proportion (44.85%) of the variations in the different plant rhizosphere microbiomes could be explained by the plant species (Supporting Information Figure S4). The results supported the hypothesis that the more phylogenetically distant the plant hosts, the more distinct their associated bacterial communities should be (Lambais, Lucheta, & Crowley, 2014; Pérez‐Jaramillo, Mendes, & Raaijmakers, 2016). In this case, the bacterial diversity and OTU abundance of the rhizosphere microbiome were significantly related to the plant taxa, at least at the species and family levels.

## CONFLICT OF INTEREST

The authors declare that they have no competing interests.

## AUTHORS CONTRIBUTION

BT and SL designed the experiments. SL, XX, BT, ZC, JX, RM, XY, and YZ carried out the metagenomic analyses. SL, XX, ZC, LZ, and BZ carried out the biochemical analyses. BT, SL XX, and ZC wrote the manuscript. All authors read the final manuscript.

## ETHICS STATEMENT

None required.

## Data Availability

The raw sequencing reads dataset was deposited at GenBank and the NCBI Short Read Archive under the project accession number PRJNA316593 and accession number SRR7012890, respectively.
