# Supplementary material for: Analysis of the community composition and bacterial diversity of the rhizosphere microbiome across different plant taxa
Source: Microbiologyopen. 2018 Nov 22;8(6):e00762. doi: 10.1002/mbo3.762 (PMC6562120; doi:10.1002/mbo3.762)

**Supporting information**

**Table S1 Experimental design**

|  |  |  |  | Sample | Sample |
| --- | --- | --- | --- | --- | --- |
| Treatments | Taxonomy of plant species | | | name | number |
| Plant | Asterales | Asteraceae | *Artemisia argyi* | Aar | 5 |
|  |  |  | *Ageratum conyzoides* | Aco | 5 |
|  |  |  | *Erigeron annuus* | Ean | 5 |
|  |  |  | *Bidens biternata* | Bbi | 5 |
|  | Malpighiales | Euphorbiaceae | *Euphorbia hirta* | Ehi | 5 |
|  |  | Violaceae | *Viola japonica* | Vja | 5 |

**Table S2 The designated experimental factors used as the examination of the significance on bacterial communities in the CAP analysis.**

| Sample ID | Species | Family | Order | life_cycle | root_system |
| --- | --- | --- | --- | --- | --- |
| Ehi1 | Ehi | Euphorbiaceae | Malpighiales | annual | taproot |
| Ehi2 | Ehi | Euphorbiaceae | Malpighiales | annual | taproot |
| Ehi3 | Ehi | Euphorbiaceae | Malpighiales | annual | taproot |
| Ehi4 | Ehi | Euphorbiaceae | Malpighiales | annual | taproot |
| Ehi5 | Ehi | Euphorbiaceae | Malpighiales | annual | taproot |
| Aco1 | Aco | Asteraceae | Asterales | annual | fibrous |
| Aco2 | Aco | Asteraceae | Asterales | annual | fibrous |
| Aco3 | Aco | Asteraceae | Asterales | annual | fibrous |
| Aco4 | Aco | Asteraceae | Asterales | annual | fibrous |
| Aco5 | Aco | Asteraceae | Asterales | annual | fibrous |
| Ean1 | Ean | Asteraceae | Asterales | biennial | fibrous |
| Ean2 | Ean | Asteraceae | Asterales | biennial | fibrous |
| Ean3 | Ean | Asteraceae | Asterales | biennial | fibrous |
| Ean4 | Ean | Asteraceae | Asterales | biennial | fibrous |
| Ean5 | Ean | Asteraceae | Asterales | biennial | fibrous |
| Bbi1 | Bbi | Asteraceae | Asterales | annual | fibrous |
| Bbi2 | Bbi | Asteraceae | Asterales | annual | fibrous |
| Bbi3 | Bbi | Asteraceae | Asterales | annual | fibrous |
| Bbi4 | Bbi | Asteraceae | Asterales | annual | fibrous |
| Bbi5 | Bbi | Asteraceae | Asterales | annual | fibrous |
| Vja1 | Vja | Violaceae | Malpighiales | perennial | taproot |
| Vja2 | Vja | Violaceae | Malpighiales | perennial | taproot |
| Vja3 | Vja | Violaceae | Malpighiales | perennial | taproot |
| Vja4 | Vja | Violaceae | Malpighiales | perennial | taproot |
| Vja5 | Vja | Violaceae | Malpighiales | perennial | taproot |
| Aar1 | Aar | Asteraceae | Asterales | perennial | taproot |
| Aar2 | Aar | Asteraceae | Asterales | perennial | taproot |
| Aar3 | Aar | Asteraceae | Asterales | perennial | taproot |
| Aar4 | Aar | Asteraceae | Asterales | perennial | taproot |
| Aar5 | Aar | Asteraceae | Asterales | perennial | taproot |

**Table S3 Summary of data and read processing steps.**

| Sample ID | Conditions | Raw paired data | Assembled data | Quality ﬁltration | Mean_len |
| --- | --- | --- | --- | --- | --- |
| Ehi1 | rhizosphere soil | 46613 | 46524 | 28246 | 414 |
| Ehi2 | rhizosphere soil | 42442 | 42370 | 25487 | 416 |
| Ehi3 | rhizosphere soil | 49169 | 49106 | 32016 | 416 |
| Ehi4 | rhizosphere soil | 42917 | 42839 | 25638 | 415 |
| Ehi5 | rhizosphere soil | 41502 | 41383 | 24895 | 414 |
| Aco1 | rhizosphere soil | 43021 | 42961 | 28399 | 414 |
| Aco2 | rhizosphere soil | 44307 | 44240 | 29822 | 416 |
| Aco3 | rhizosphere soil | 45649 | 45576 | 30586 | 416 |
| Aco4 | rhizosphere soil | 42115 | 42039 | 28035 | 415 |
| Aco5 | rhizosphere soil | 48548 | 48452 | 28994 | 415 |
| Ean1 | rhizosphere soil | 40555 | 40489 | 27963 | 416 |
| Ean2 | rhizosphere soil | 42311 | 42237 | 25430 | 416 |
| Ean3 | rhizosphere soil | 41689 | 41634 | 26935 | 415 |
| Ean4 | rhizosphere soil | 44748 | 44666 | 26965 | 415 |
| Ean5 | rhizosphere soil | 42861 | 42773 | 24445 | 415 |
| Bbi1 | rhizosphere soil | 41517 | 41474 | 29083 | 413 |
| Bbi2 | rhizosphere soil | 42302 | 42228 | 26737 | 412 |
| Bbi3 | rhizosphere soil | 39086 | 39020 | 25947 | 414 |
| Bbi4 | rhizosphere soil | 43850 | 43770 | 27523 | 412 |
| Bbi5 | rhizosphere soil | 41436 | 41364 | 28446 | 412 |
| Vja1 | rhizosphere soil | 31302 | 31277 | 20850 | 415 |
| Vja2 | rhizosphere soil | 40501 | 40465 | 25950 | 414 |
| Vja3 | rhizosphere soil | 41674 | 41637 | 30362 | 414 |
| Vja4 | rhizosphere soil | 38179 | 38118 | 27225 | 414 |
| Vja5 | rhizosphere soil | 40620 | 40537 | 26021 | 412 |
| Aar1 | rhizosphere soil | 43987 | 43930 | 29422 | 414 |
| Aar2 | rhizosphere soil | 43225 | 43163 | 28958 | 414 |
| Aar3 | rhizosphere soil | 41713 | 41655 | 28412 | 414 |
| Aar4 | rhizosphere soil | 37334 | 37276 | 24592 | 413 |
| Aar5 | rhizosphere soil | 42404 | 42333 | 29099 | 412 |

**Table S4** Summary of Species richness estimator and alpha diversity index.

| Sample ID | Nseqs | Num_otus | Coverage | ACE | Chao1 | Simpson | Shannon |
| --- | --- | --- | --- | --- | --- | --- | --- |
| Ehi1 | 20850 | 2560 | 0.9528 | 3708.29 | 3663.92 | 0.9938 | 9.265 |
| Ehi2 | 20850 | 2550 | 0.9538 | 3643.86 | 3710.42 | 0.9955 | 9.462 |
| Ehi3 | 20850 | 2642 | 0.9550 | 3667.65 | 3601.50 | 0.9963 | 9.646 |
| Ehi4 | 20850 | 2338 | 0.9573 | 3394.71 | 3511.90 | 0.9950 | 9.265 |
| Ehi5 | 20850 | 2272 | 0.9585 | 3293.92 | 3371.06 | 0.9944 | 9.133 |
| Aco1 | 20850 | 2990 | 0.9456 | 4317.70 | 4168.65 | 0.9957 | 9.806 |
| Aco2 | 20850 | 3255 | 0.9395 | 4743.98 | 4688.68 | 0.9973 | 10.122 |
| Aco3 | 20850 | 2860 | 0.9485 | 4088.21 | 4019.53 | 0.9959 | 9.732 |
| Aco4 | 20850 | 2576 | 0.9581 | 3482.93 | 3463.24 | 0.9970 | 9.742 |
| Aco5 | 20850 | 3034 | 0.9469 | 4250.10 | 4178.18 | 0.9974 | 10.038 |
| Ean1 | 20850 | 2602 | 0.9561 | 3580.97 | 3595.24 | 0.9941 | 9.540 |
| Ean2 | 20850 | 3049 | 0.9438 | 4395.33 | 4497.28 | 0.9971 | 9.957 |
| Ean3 | 20850 | 3059 | 0.9451 | 4332.35 | 4316.30 | 0.9961 | 9.888 |
| Ean4 | 20850 | 2767 | 0.9516 | 3899.38 | 3858.10 | 0.9960 | 9.726 |
| Ean5 | 20850 | 2761 | 0.9542 | 3763.19 | 3726.12 | 0.9967 | 9.814 |
| Bbi1 | 20850 | 2509 | 0.9579 | 3485.44 | 3509.01 | 0.9964 | 9.669 |
| Bbi2 | 20850 | 2717 | 0.9517 | 3887.77 | 3813.37 | 0.9965 | 9.745 |
| Bbi3 | 20850 | 2826 | 0.9524 | 3892.05 | 3871.82 | 0.9976 | 9.983 |
| Bbi4 | 20850 | 2789 | 0.9542 | 3763.92 | 3794.71 | 0.9973 | 9.967 |
| Bbi5 | 20850 | 2741 | 0.9527 | 3826.87 | 3789.69 | 0.9965 | 9.785 |
| Vja1 | 20850 | 2941 | 0.9475 | 4162.91 | 4104.04 | 0.9971 | 9.901 |
| Vja2 | 20850 | 2716 | 0.9482 | 4061.53 | 4086.96 | 0.9966 | 9.661 |
| Vja3 | 20850 | 2388 | 0.9616 | 3227.33 | 3195.05 | 0.9969 | 9.616 |
| Vja4 | 20850 | 3137 | 0.9452 | 4359.49 | 4419.50 | 0.9978 | 10.171 |
| Vja5 | 20850 | 2726 | 0.9512 | 3859.71 | 3849.12 | 0.9968 | 9.734 |
| Aar1 | 20850 | 3071 | 0.9479 | 4205.53 | 4146.12 | 0.9964 | 10.026 |
| Aar2 | 20850 | 3275 | 0.9440 | 4480.85 | 4428.18 | 0.9971 | 10.184 |
| Aar3 | 20850 | 3247 | 0.9444 | 4450.04 | 4410.02 | 0.9971 | 10.185 |
| Aar4 | 20850 | 3036 | 0.9473 | 4225.40 | 4123.10 | 0.9966 | 9.948 |
| Aar5 | 20850 | 3137 | 0.9434 | 4444.60 | 4442.08 | 0.9964 | 10.000 |

**Table S5. Taxonomic identification of the most 100 abundant core OTUs in six plant species.**

| **#OTU ID** | **Aar** | **Aco** | **Bbi** | **Ean** | **Ehi** | **Vja** | **Taxonomy** |
| --- | --- | --- | --- | --- | --- | --- | --- |
| OTU_3 | 215.6 | 501.8 | 532.2 | 617.2 | 438.6 | 148.8 | Proteobacteria; Sphingomonadales; Sphingomonas |
| OTU_1 | 580.8 | 161.4 | 429.8 | 158.4 | 156.2 | 386.4 | Proteobacteria; Rhizobiales; Bradyrhizobium |
| OTU_30 | 157.6 | 349.2 | 143.8 | 362.6 | 334 | 139.8 | Proteobacteria; Nitrosomonadales; uncultured |
| OTU_2 | 600.2 | 173.6 | 119.4 | 102.6 | 176.8 | 241.8 | Proteobacteria; Rhizobiales; Variibacter |
| OTU_4 | 53.4 | 373.8 | 61 | 405.8 | 114.8 | 86.6 | Bacteroidetes; Sphingobacteriales; uncultured |
| OTU_25 | 58.2 | 170 | 112.2 | 212.8 | 485.8 | 17 | Acidobacteria; Subgroup 4; uncultured bacterium |
| OTU_14 | 141.2 | 75.2 | 259.2 | 96.2 | 18.6 | 204.8 | Proteobacteria; Rhizobiales; Microvirga |
| OTU_53 | 127.8 | 85.4 | 154.6 | 81.6 | 64 | 221.2 | Proteobacteria; Xanthomonadales; Steroidobacter |
| OTU_5 | 35.8 | 63.2 | 4.2 | 16 | 596.6 | 4.8 | Bacteroidetes; Sphingobacteriales; Ferruginibacter |
| OTU_13 | 94.4 | 112.8 | 132.2 | 126.6 | 85.4 | 127.4 | Proteobacteria; Burkholderiales; Variovorax |
| OTU_29 | 76.8 | 156.6 | 31.6 | 180.8 | 15 | 202.6 | Proteobacteria; Sphingomonadales; Sphingomonas |
| OTU_58 | 146.6 | 51.8 | 99 | 59.8 | 17.4 | 268.6 | Actinobacteria; Corynebacteriales; uncultured |
| OTU_49 | 78.2 | 96.8 | 207.8 | 111.8 | 41.6 | 90.4 | Proteobacteria; Xanthomonadales; Acidibacter |
| OTU_135 | 45.8 | 149 | 56.4 | 131.8 | 164.4 | 76 | Proteobacteria; Nitrosomonadales; uncultured |
| OTU_48 | 105.8 | 70 | 122.4 | 59 | 54.2 | 151.6 | Chloroflexi |
| OTU_37 | 180.2 | 33.8 | 116.4 | 25 | 113.6 | 38.2 | Verrucomicrobia; Chthoniobacterales; uncultured |
| OTU_84 | 132.8 | 55.2 | 68.8 | 61.6 | 58 | 95.8 | Proteobacteria; Rhizobiales |
| OTU_7 | 105.6 | 102.6 | 32.4 | 63.2 | 18 | 124.2 | Proteobacteria; Rhizobiales; Rhizobium |
| OTU_21 | 54.6 | 103.6 | 33.8 | 90.4 | 2.2 | 148.8 | Bacteroidetes; Cytophagales; Chryseolinea |
| OTU_95 | 29.4 | 82.8 | 42.6 | 50.2 | 211 | 13.2 | Acidobacteria; Subgroup 4; uncultured bacterium |
| OTU_11 | 152.4 | 41.8 | 0.8 | 43 | 0.2 | 186 | Actinobacteria; Corynebacteriales |
| OTU_63 | 40 | 51.6 | 87.4 | 57.8 | 158 | 24.4 | Bacteroidetes; Sphingobacteriales |
| OTU_7048 | 71 | 65.4 | 61 | 41.4 | 147 | 31.2 | Proteobacteria; Nitrosomonadales; uncultured |
| OTU_142 | 47.2 | 100 | 37.6 | 68.6 | 131.4 | 28.8 | Acidobacteria; Subgroup 6; uncultured bacterium |
| OTU_59 | 97.6 | 49 | 94.8 | 41.6 | 23.2 | 88.2 | Proteobacteria; Myxococcales; uncultured |
| OTU_6 | 25.2 | 117 | 0.6 | 181.6 | 6.8 | 62.6 | Proteobacteria; Sphingomonadales; Sphingomonas |
| OTU_44 | 45.4 | 71.6 | 18.2 | 70.4 | 52.8 | 133.2 | Proteobacteria; Pseudomonadales; uncultured |
| OTU_8 | 103.8 | 59.2 | 45.8 | 23.2 | 135.8 | 20.6 | Planctomycetes; WD2101 soil group; uncultured |
| OTU_96 | 31.4 | 57.8 | 74.4 | 132.2 | 41.6 | 50.6 | Proteobacteria; Burkholderiales; Variovorax |
| OTU_50 | 51 | 43.6 | 7.2 | 13.2 | 263.6 | 9 | Acidobacteria; Acidobacteriales; uncultured |
| OTU_24 | 51 | 70 | 53.6 | 97.4 | 61.2 | 42.2 | Chloroflexi; Anaerolineales; uncultured |
| OTU_27 | 93.2 | 39.4 | 74 | 29.2 | 35.6 | 99 | Actinobacteria; Acidimicrobiales; uncultured |
| OTU_102 | 88.2 | 39.8 | 56.2 | 36.8 | 55.8 | 78.6 | Proteobacteria; Rhizobiales; uncultured |
| OTU_55 | 50.2 | 94 | 23.8 | 81.4 | 52.2 | 48 | Proteobacteria; Nitrosomonadales; uncultured |
| OTU_475 | 64 | 44.8 | 49.4 | 20.6 | 132.4 | 36.2 | Acidobacteria; Subgroup 3; Candidatus Solibacter |
| OTU_133 | 58.8 | 42.4 | 121.2 | 50.4 | 24.2 | 49.2 | Acidobacteria; Subgroup 3; Bryobacter |
| OTU_143 | 57.8 | 66 | 40.2 | 51.8 | 92.8 | 32.8 | Proteobacteria; Rhizobiales; Rhizomicrobium |
| OTU_374 | 40 | 69.2 | 30.6 | 81.2 | 81.8 | 34.2 | Proteobacteria; Nitrosomonadales; uncultured |
| OTU_42 | 70 | 36.2 | 98.4 | 41 | 6.2 | 82.6 | Proteobacteria; Rhodospirillales; Skermanella |
| OTU_10 | 18.2 | 92.4 | 17.8 | 25 | 165.6 | 14.2 | Acidobacteria; Subgroup 4; uncultured bacterium |
| OTU_12 | 16.4 | 107.8 | 23.2 | 127.6 | 13.4 | 32.8 | Bacteroidetes; Sphingobacteriales; uncultured |
| OTU_9 | 67.4 | 52.2 | 5.6 | 51.6 | 3.6 | 123.6 | Proteobacteria; Myxococcales; uncultured |
| OTU_17 | 5.4 | 7.4 | 9 | 194.2 | 81.4 | 1 | Acidobacteria; Subgroup 4; uncultured bacterium |
| OTU_90 | 63.6 | 42.2 | 52.4 | 36.8 | 58.4 | 44.4 | Proteobacteria; Rhizobiales; Variibacter |
| OTU_26 | 84 | 42.2 | 72.8 | 19.4 | 37 | 41 | Acidobacteria; Subgroup 6; uncultured bacterium |
| OTU_108 | 13.8 | 108.8 | 2.8 | 94 | 6.6 | 67 | Proteobacteria; Pseudomonadales; Pseudomonas |
| OTU_32 | 29.8 | 55.8 | 101.2 | 39 | 21.8 | 44.4 | Bacteroidetes; Sphingobacteriales |
| OTU_136 | 18.4 | 46.6 | 108.2 | 65 | 35.4 | 15.6 | Bacteroidetes; Sphingobacteriales; Flavisolibacter |
| OTU_2340 | 42.8 | 30 | 91.4 | 32.4 | 48 | 41 | Proteobacteria; Rhizobiales; Rhodoplanes |
| OTU_46 | 86.2 | 25.6 | 51 | 31.8 | 15 | 72.4 | Actinobacteria; Gaiellales; uncultured bacterium |
| OTU_89 | 43 | 31 | 93.4 | 28.8 | 76.4 | 8 | Chloroflexi; Anaerolineales; uncultured |
| OTU_114 | 71.4 | 43.4 | 12.8 | 21 | 114.8 | 17.2 | Acidobacteria; Subgroup 5; uncultured bacterium |
| OTU_5356 | 12.2 | 24 | 19.8 | 19.6 | 194.4 | 5.2 | Acidobacteria; Subgroup 4; uncultured bacterium |
| OTU_15 | 43 | 25.4 | 5.8 | 26.4 | 0.2 | 172 | Actinobacteria; Acidimicrobiales; uncultured |
| OTU_43 | 35.2 | 27.4 | 23.2 | 20 | 136.6 | 28.8 | Proteobacteria; Nitrosomonadales; uncultured |
| OTU_590 | 28 | 52.2 | 31 | 70.4 | 35.2 | 52.6 | Proteobacteria; Burkholderiales; uncultured |
| OTU_56 | 63.6 | 25.4 | 6.4 | 15.8 | 139.6 | 16.6 | Verrucomicrobia; Chthoniobacterales; uncultured |
| OTU_75 | 50.6 | 67.8 | 17.8 | 61 | 36.6 | 31.8 | Bacteroidetes; Cytophagales; Adhaeribacter |
| OTU_47 | 23 | 13.2 | 32.4 | 7.2 | 180.6 | 8.8 | Gemmatimonadetes; Gemmatimonadales |
| OTU_60 | 34 | 70.6 | 32.4 | 40.4 | 69 | 14.8 | Proteobacteria; SC-I-84; uncultured bacterium |
| OTU_34 | 40.6 | 35.6 | 87.4 | 62.4 | 15.4 | 19.8 | Actinobacteria; Micrococcales; Arthrobacter |
| OTU_20 | 32.2 | 79.4 | 29 | 66.6 | 26 | 27.6 | Proteobacteria; Rhodospirillales; uncultured |
| OTU_231 | 26.8 | 72.8 | 32.8 | 77.6 | 35.2 | 14.2 | Proteobacteria; Burkholderiales; Achromobacter |
| OTU_127 | 39.8 | 38.8 | 65.6 | 31.8 | 24.6 | 56.6 | Proteobacteria; Rhizobiales; Nordella |
| OTU_267 | 20 | 97.8 | 18.4 | 71.4 | 11.2 | 38 | Proteobacteria; Xanthomonadales; Arenimonas |
| OTU_39 | 14.6 | 73 | 11.2 | 42 | 104.4 | 3.4 | Acidobacteria; Subgroup 4; Blastocatella |
| OTU_321 | 44.2 | 22 | 51.4 | 32 | 16.4 | 76.8 | Proteobacteria; Rhizobiales; Mesorhizobium |
| OTU_120 | 30 | 48 | 44.8 | 51.4 | 31.4 | 36 | Proteobacteria; Caulobacterales; Phenylobacterium |
| OTU_483 | 47 | 21 | 75.8 | 26.6 | 24.6 | 44.6 | Actinobacteria; Solirubrobacterales; Solirubrobacter |
| OTU_79 | 75 | 14.4 | 46 | 12.6 | 54.6 | 36.8 | Actinobacteria; Gaiellales; uncultured bacterium |
| OTU_19 | 51.4 | 34.6 | 50.4 | 20.6 | 40.2 | 36 | Proteobacteria; Rhodospirillales; uncultured |
| OTU_54 | 23 | 9.8 | 69.6 | 12.4 | 104.4 | 13.8 | Firmicutes; Bacillales; Bacillus |
| OTU_72 | 17.2 | 75 | 27 | 76 | 6.6 | 30.6 | Proteobacteria; Sphingomonadales |
| OTU_190 | 49.8 | 39.8 | 28.8 | 36 | 30.2 | 42.2 | Proteobacteria; Rhodospirillales; Reyranella |
| OTU_192 | 40.6 | 38 | 52.6 | 18.6 | 55 | 21.6 | Proteobacteria; Nitrosomonadales; uncultured |
| OTU_117 | 20.6 | 52 | 37 | 64.6 | 6.6 | 45.4 | Acidobacteria; Subgroup 6; uncultured bacterium |
| OTU_33 | 5 | 53.2 | 10.6 | 119.6 | 1.4 | 35.6 | Proteobacteria; Xanthomonadales; uncultured |
| OTU_36 | 15.6 | 86.4 | 22.2 | 66.4 | 22.4 | 10.4 | Acidobacteria; Subgroup 4; Blastocatella |
| OTU_76 | 62.6 | 14.2 | 50.4 | 12.4 | 65.6 | 17.8 | Verrucomicrobia; Chthoniobacterales; uncultured |
| OTU_315 | 46.2 | 25.4 | 60.4 | 34.4 | 9.6 | 46.2 | Actinobacteria; Propionibacteriales; Marmoricola |
| OTU_83 | 26.8 | 48 | 6.4 | 29.4 | 3.8 | 107.6 | Proteobacteria; Xanthomonadales; uncultured |
| OTU_64 | 27.6 | 48 | 41.8 | 71.2 | 6.4 | 25.4 | Bacteroidetes; Sphingobacteriales |
| OTU_23 | 28.2 | 27.4 | 1.4 | 24.8 | 1 | 135.6 | Actinobacteria; Acidimicrobiales; Illumatobacter |
| OTU_69 | 7.2 | 15 | 8.2 | 17.2 | 167.4 | 2.2 | Acidobacteria; Subgroup 4; uncultured bacterium |
| OTU_147 | 56.6 | 22.8 | 37.2 | 24.2 | 32.8 | 41.8 | Proteobacteria; Rhizobiales; Pedomicrobium |
| OTU_229 | 64 | 37.6 | 25.6 | 28.2 | 38.8 | 21 | Proteobacteria; Myxococcales; Haliangium |
| OTU_144 | 29.2 | 54.6 | 30 | 47 | 46.4 | 7.8 | Acidobacteria; Subgroup 4; Blastocatella |
| OTU_163 | 51 | 34 | 16.6 | 25.6 | 49.6 | 35.2 | Acidobacteria; Subgroup 6; uncultured bacterium |
| OTU_35 | 8.6 | 48 | 26.6 | 86.8 | 27.8 | 14 | Acidobacteria; Subgroup 4; Blastocatella |
| OTU_150 | 39 | 15 | 68 | 23.8 | 13.4 | 52.6 | Actinobacteria; Gaiellales; Gaiella |
| OTU_52 | 31.8 | 47 | 23.2 | 44 | 22.2 | 43.2 | Proteobacteria; Xanthomonadales; uncultured |
| OTU_16 | 50.4 | 51.6 | 19.8 | 32 | 21 | 36 | Nitrospirae; Nitrospirales; Nitrospira |
| OTU_94 | 25.4 | 61.8 | 7.4 | 67.2 | 24.4 | 23.6 | Proteobacteria; Enterobacteriales; Enterobacter |
| OTU_70 | 34.8 | 61.2 | 14 | 49.2 | 13.4 | 36.2 | Proteobacteria |
| OTU_240 | 40 | 14.2 | 61 | 20.4 | 1.2 | 70.2 | Actinobacteria; Micrococcales; Cellulosimicrobium |
| OTU_920 | 11.8 | 15.8 | 18.2 | 18.6 | 140 | 1.6 | Bacteroidetes; Sphingobacteriales; Ferruginibacter |
| OTU_62 | 74 | 17 | 45.2 | 8.4 | 31.4 | 28.6 | Chloroflexi; Chloroflexales; Roseiflexus |
| OTU_92 | 50.2 | 43.4 | 45.4 | 34.4 | 17 | 14 | Verrucomicrobia; Chthoniobacterales; uncultured |
| OTU_169 | 42 | 15.8 | 49 | 39.8 | 39.4 | 18 | Planctomycetes; Planctomycetales; Pirellula |
| OTU_97 | 41.8 | 31.6 | 68.2 | 40.2 | 12.8 | 8.6 | Cyanobacteria |

**Table S6. Taxonomic identification of the most 100 differentially abundant OTUs.**

| **OUT ID** | **Test-Statistic** | **P** | **FDR_P** | **Bonferroni_P** | **Taxonomy** |
| --- | --- | --- | --- | --- | --- |
| OTU_3 | 18.5894 | 1.48E-07 | 5.48E-06 | 0.0012 | Alphaproteobacteria; Sphingomonadales |
| OTU_1 | 17.4280 | 2.67E-07 | 8.43E-06 | 0.0022 | Alphaproteobacteria; Rhizobiales |
| OTU_31 | 21.4920 | 3.77E-08 | 2.04E-06 | 0.0003 | Betaproteobacteria; Nitrosomonadales |
| OTU_2 | 13.7546 | 2.16E-06 | 4.03E-05 | 0.0175 | Alphaproteobacteria; Rhizobiales |
| OTU_4 | 26.2234 | 5.42E-09 | 5.61E-07 | 0.0000 | Sphingobacteriia; Sphingobacteriales |
| OTU_52 | 9.3621 | 4.75E-05 | 0.000444 | 0.3834 | Gammaproteobacteria; Xanthomonadales |
| OTU_27 | 24.5062 | 1.06E-08 | 8.45E-07 | 0.0001 | Acidobacteria; Subgroup 4 |
| OTU_14 | 26.1525 | 5.56E-09 | 5.69E-07 | 0.0000 | Alphaproteobacteria; Rhizobiales |
| OTU_134 | 23.0610 | 1.91E-08 | 1.30E-06 | 0.0002 | Betaproteobacteria; Nitrosomonadales |
| OTU_48 | 5.7881 | 0.001213 | 0.006014 | 1.0000 | Gammaproteobacteria; Xanthomonadales |
| OTU_49 | 6.1037 | 0.000879 | 0.004789 | 1.0000 | KD4-96 |
| OTU_83 | 5.3875 | 0.001846 | 0.008455 | 1.0000 | Alphaproteobacteria; Rhizobiales |
| OTU_57 | 23.5277 | 1.57E-08 | 1.12E-06 | 0.0001 | Actinobacteria; Corynebacteriales |
| OTU_28 | 10.5161 | 1.95E-05 | 0.000213 | 0.1575 | Alphaproteobacteria; Sphingomonadales |
| OTU_36 | 9.8262 | 3.30E-05 | 0.000329 | 0.2660 | Spartobacteria; Chthoniobacterales |
| OTU_147 | 14.5840 | 1.31E-06 | 2.80E-05 | 0.0105 | Acidobacteria; Subgroup 6 |
| OTU_100 | 5.9140 | 0.001066 | 0.005410 | 1.0000 | Alphaproteobacteria; Rhizobiales |
| OTU_61 | 12.7067 | 4.23E-06 | 6.70E-05 | 0.0341 | Sphingobacteriia; Sphingobacteriales |
| OTU_59 | 13.0824 | 3.31E-06 | 5.56E-05 | 0.0267 | Deltaproteobacteria; Myxococcales |
| OTU_7 | 9.4622 | 4.39E-05 | 0.000416 | 0.3540 | Alphaproteobacteria; Rhizobiales |
| OTU_7475 | 5.7900 | 0.001211 | 0.006014 | 1.0000 | Betaproteobacteria; Nitrosomonadales |
| OTU_25 | 9.3115 | 4.95E-05 | 0.000460 | 0.3992 | Acidimicrobiia; Acidimicrobiales |
| OTU_95 | 4.2628 | 0.006469 | 0.022648 | 1.0000 | Betaproteobacteria; Burkholderiales |
| OTU_55 | 4.1975 | 0.006983 | 0.024165 | 1.0000 | Betaproteobacteria; Nitrosomonadales |
| OTU_131 | 16.0190 | 5.72E-07 | 1.51E-05 | 0.0046 | Acidobacteria; Subgroup 3 |
| OTU_143 | 3.6769 | 0.013028 | 0.039304 | 1.0000 | Alphaproteobacteria; Rhizobiales |
| OTU_44 | 4.8041 | 0.003488 | 0.013939 | 1.0000 | Gammaproteobacteria; Pseudomonadales |
| OTU_89 | 3.5673 | 0.014907 | 0.043629 | 1.0000 | Alphaproteobacteria; Rhizobiales |
| OTU_476 | 8.6430 | 0.000086 | 0.000708 | 0.6906 | Acidobacteria; Subgroup 3 |
| OTU_371 | 2.8618 | 0.036451 | 0.089634 | 1.0000 | Betaproteobacteria; Nitrosomonadales |
| OTU_21 | 64.8536 | 3.77E-13 | 3.80E-10 | 0.0000 | Cytophagia; Cytophagales |
| OTU_8 | 9.4854 | 4.31E-05 | 0.0004113 | 0.3476 | Phycisphaerae; WD2101 soil group |
| OTU_26 | 8.5289 | 9.42E-05 | 0.0007624 | 0.7601 | Acidobacteria; Subgroup 6 |
| OTU_135 | 23.9822 | 1.31E-08 | 9.95E-07 | 0.0001 | Sphingobacteriia; Sphingobacteriales |
| OTU_589 | 6.0657 | 0.000914 | 0.004912 | 1.0000 | Betaproteobacteria; Burkholderiales |
| OTU_2427 | 11.2553 | 1.14E-05 | 0.0001440 | 0.0920 | Alphaproteobacteria; Rhizobiales |
| OTU_42 | 22.0304 | 2.97E-08 | 1.73E-06 | 0.0002 | Alphaproteobacteria; Rhodospirillales |
| OTU_126 | 6.9046 | 0.000402 | 0.002522 | 1.0000 | Alphaproteobacteria; Rhizobiales |
| OTU_94 | 10.6410 | 1.78E-05 | 0.000200 | 0.1436 | Acidobacteria; Subgroup 4 |
| OTU_20 | 13.0670 | 3.34E-06 | 5.58E-05 | 0.0270 | Alphaproteobacteria; Rhodospirillales |
| OTU_46 | 17.5547 | 2.50E-07 | 8.08E-06 | 0.0020 | Thermoleophilia; Gaiellales |
| OTU_32 | 4.1884 | 0.007058 | 0.024361 | 1.0000 | Sphingobacteriia; Sphingobacteriales |
| OTU_74 | 3.4814 | 0.016578 | 0.047340 | 1.0000 | Cytophagia; Cytophagales |
| OTU_58 | 12.1988 | 5.93E-06 | 8.73E-05 | 0.0478 | Betaproteobacteria; SC-I-84 |
| OTU_327 | 29.9657 | 1.42E-09 | 1.88E-07 | 0.0000 | Alphaproteobacteria; Rhizobiales |
| OTU_19 | 8.8903 | 6.96E-05 | 0.000606 | 0.5623 | Alphaproteobacteria; Rhodospirillales |
| OTU_491 | 8.7523 | 7.81E-05 | 0.000663 | 0.6303 | Thermoleophilia; Solirubrobacterales |
| OTU_192 | 12.0223 | 6.68E-06 | 9.53E-05 | 0.0539 | Betaproteobacteria; Nitrosomonadales |
| OTU_144 | 3.9313 | 0.009575 | 0.030994 | 1.0000 | Alphaproteobacteria; Rhizobiales |
| OTU_112 | 20.9961 | 4.71E-08 | 2.33E-06 | 0.0004 | Acidobacteria; Subgroup 5 |
| OTU_196 | 18.5987 | 1.47E-07 | 5.48E-06 | 0.0012 | OPB35 soil group; uncultured bacterium |
| OTU_77 | 21.8965 | 3.15E-08 | 1.81E-06 | 0.0003 | Acidimicrobiia; Acidimicrobiales |
| OTU_11 | 12.5028 | 4.83E-06 | 7.41E-05 | 0.0390 | Sphingobacteriia; Sphingobacteriales |
| OTU_53 | 2.6341 | 0.049117 | 0.109840 | 1.0000 | Gammaproteobacteria; Xanthomonadales |
| OTU_313 | 12.8980 | 3.73E-06 | 6.10E-05 | 0.0301 | Actinobacteria; Propionibacteriales |
| OTU_160 | 8.6636 | 8.41E-05 | 0.000698 | 0.6787 | Acidobacteria; Subgroup 6 |
| OTU_10 | 21.3604 | 4.00E-08 | 2.14E-06 | 0.0003 | Acidobacteria; Subgroup 4 |
| OTU_88 | 6.6681 | 0.000504 | 0.003043 | 1.0000 | Anaerolineae; Anaerolineales |
| OTU_232 | 5.0759 | 0.002584 | 0.010926 | 1.0000 | Betaproteobacteria; Burkholderiales |
| OTU_16 | 4.5928 | 0.004425 | 0.016794 | 1.0000 | Nitrospira; Nitrospirales |
| OTU_78 | 12.4685 | 4.95E-06 | 7.55E-05 | 0.0399 | Thermoleophilia; Gaiellales |
| OTU_265 | 13.0546 | 3.37E-06 | 5.61E-05 | 0.0272 | Gammaproteobacteria; Xanthomonadales |
| OTU_315 | 13.2247 | 3.02E-06 | 5.22E-05 | 0.0244 | Actinobacteria; Streptomycetales |
| OTU_142 | 10.3612 | 2.19E-05 | 0.000234 | 0.1769 | Acidobacteria; Subgroup 4 |
| OTU_226 | 2.7185 | 0.043957 | 0.104309 | 1.0000 | Deltaproteobacteria; Myxococcales |
| OTU_68 | 11.9900 | 6.83E-06 | 9.61E-05 | 0.0551 | Betaproteobacteria |
| OTU_43 | 9.7048 | 3.62E-05 | 0.000356 | 0.2924 | Betaproteobacteria; Nitrosomonadales |
| OTU_119 | 19.4788 | 9.56E-08 | 3.98E-06 | 0.0008 | Acidobacteria; Subgroup 6 |
| OTU_33 | 3.4521 | 0.017194 | 0.048773 | 1.0000 | Actinobacteria; Micrococcales |
| OTU_146 | 10.5831 | 1.86E-05 | 0.000207 | 0.1499 | Thermoleophilia; Gaiellales |
| OTU_484 | 5.6730 | 0.001367 | 0.006612 | 1.0000 | Alphaproteobacteria; Rhizobiales |
| OTU_37 | 20.8556 | 5.02E-08 | 2.44E-06 | 0.0004 | Acidobacteria; Subgroup 4 |
| OTU_71 | 25.8194 | 6.32E-09 | 5.88E-07 | 0.0001 | Alphaproteobacteria; Sphingomonadales |
| OTU_128 | 3.3086 | 0.020577 | 0.056198 | 1.0000 | Sphingobacteriia; Sphingobacteriales |
| OTU_1701 | 6.6509 | 0.000513 | 0.003083 | 1.0000 | Betaproteobacteria; Burkholderiales |
| OTU_75 | 10.1521 | 2.57E-05 | 0.000267 | 0.2071 | Spartobacteria; Chthoniobacterales |
| OTU_170 | 3.1676 | 0.024598 | 0.064795 | 1.0000 | Planctomycetacia; Planctomycetales |
| OTU_35 | 13.1029 | 3.27E-06 | 5.50E-05 | 0.0264 | Acidobacteria; Subgroup 4 |
| OTU_155 | 11.8346 | 7.60E-06 | 0.000105 | 0.0613 | Acidimicrobiia; Acidimicrobiales |
| OTU_54 | 23.0102 | 1.95E-08 | 1.30E-06 | 0.0002 | Bacilli; Bacillales |
| OTU_92 | 3.0624 | 0.028134 | 0.072103 | 1.0000 | Spartobacteria; Chthoniobacterales |
| OTU_64 | 5.3852 | 0.001851 | 0.008470 | 1.0000 | Bacilli; Bacillales |
| OTU_45 | 21.9626 | 3.06E-08 | 1.77E-06 | 0.0002 | Actinobacteria; Frankiales |
| OTU_62 | 13.2288 | 3.01E-06 | 5.22E-05 | 0.0243 | Chloroflexia; Chloroflexales |
| OTU_60 | 24.4432 | 1.08E-08 | 8.58E-07 | 0.0001 | Alphaproteobacteria; Rhizobiales |
| OTU_220 | 17.2559 | 2.92E-07 | 8.96E-06 | 0.0024 | Thermoleophilia; Solirubrobacterales |
| OTU_63 | 16.0194 | 5.71E-07 | 1.51E-05 | 0.0046 | Sphingobacteriia; Sphingobacteriales |
| OTU_224 | 13.6454 | 2.32E-06 | 4.24E-05 | 0.0187 | Actinobacteria; Propionibacteriales |
| OTU_114 | 7.7781 | 0.000180 | 0.001313 | 1.0000 | Acidobacteria; Subgroup 4 |
| OTU_98 | 4.5653 | 0.004565 | 0.017135 | 1.0000 | Chloroplast |
| OTU_39 | 23.6377 | 1.50E-08 | 1.11E-06 | 0.0001 | Acidobacteria; Subgroup 4 |
| OTU_30 | 4.9099 | 0.003101 | 0.012669 | 1.0000 | Sphingobacteriia; Sphingobacteriales |
| OTU_264 | 7.6381 | 0.000205 | 0.001437 | 1.0000 | Acidimicrobiia; Acidimicrobiales |
| OTU_9 | 10.4050 | 2.12E-05 | 0.000228 | 0.1711 | Deltaproteobacteria; Myxococcales |
| OTU_73 | 5.5956 | 0.001482 | 0.007101 | 1.0000 | Betaproteobacteria; Nitrosomonadales |
| OTU_93 | 12.0950 | 6.36E-06 | 9.25E-05 | 0.0513 | Acidimicrobiia; Acidimicrobiales |
| OTU_102 | 21.1306 | 4.43E-08 | 2.29E-06 | 0.0004 | MB-A2-108; uncultured bacterium |
| OTU_41 | 12.7685 | 4.06E-06 | 6.49E-05 | 0.0328 | Deltaproteobacteria; Desulfurellales |
| OTU_301 | 11.0949 | 1.28E-05 | 0.0001575 | 0.1032 | OPB35 soil group; uncultured bacterium |
| OTU_513 | 12.8877 | 3.75E-06 | 6.12E-05 | 0.0303 | Opitutae; Opitutales |

**Fig. S1 The composition and relative abundance of major bacterial phyla (A) and Orders (B) in rhizosphere soil of six plant species.** Each bar represents the value of each replicate of the sample. Aco, *Ageratum conyzoides*; Ean, *Erigeron annuus*; Bbi, *Bidens biternata*; Aar, *Artemisia argyi*; Vja, *Viola japonica*; Ehi, *Euphorbia hirta*.


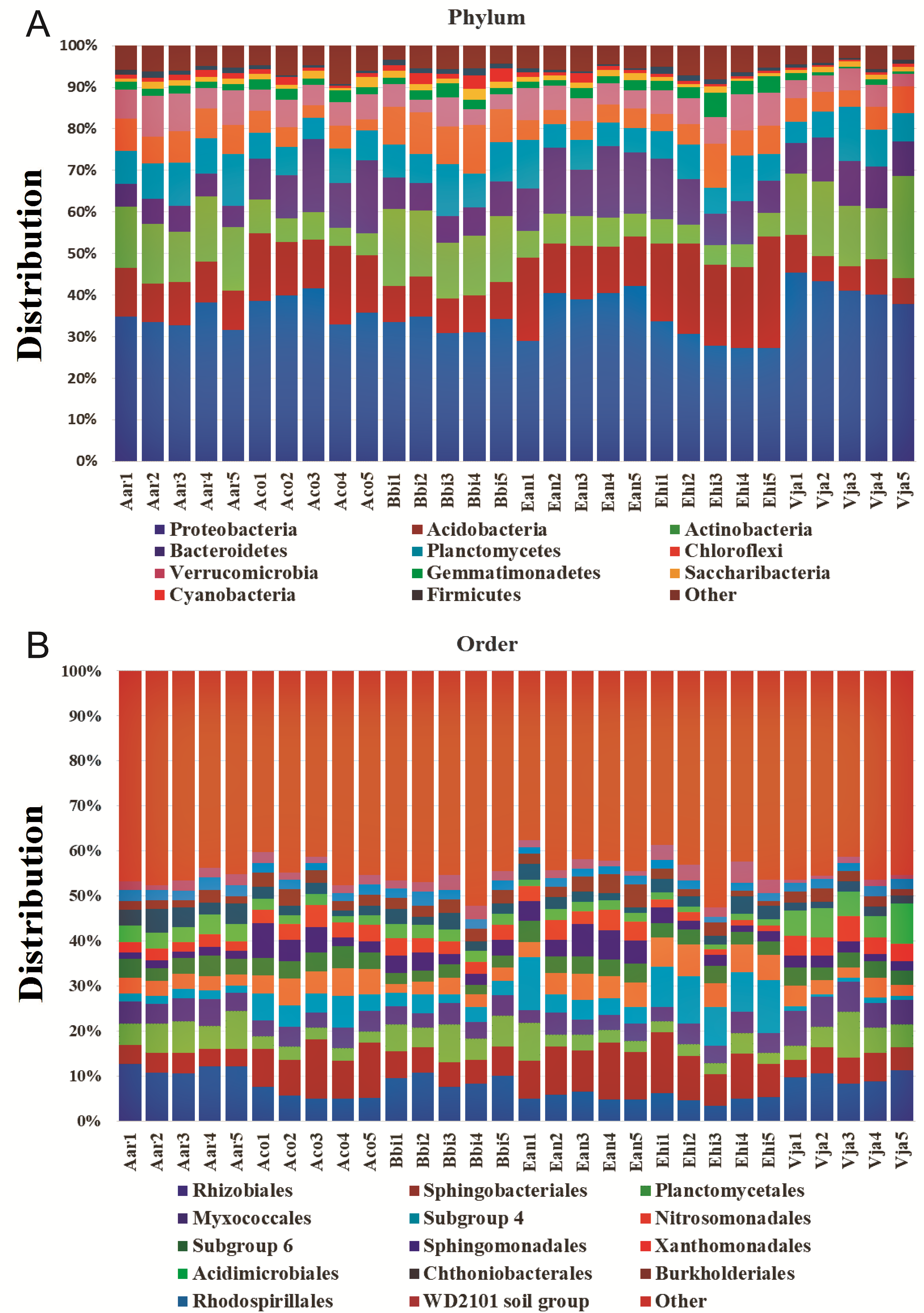


**Fig. S2 Rarefaction curves of the microbiome in rhizosphere soil of six plant species at 3% distance cutoff.** Each bar represents the average value of five replicates of each sample group. Aco, *Ageratum conyzoides*; Ean, *Erigeron annuus*; Bbi, *Bidens biternata*; Aar, *Artemisia argyi*; Vja, *Viola japonica*; Ehi, *Euphorbia hirta*.


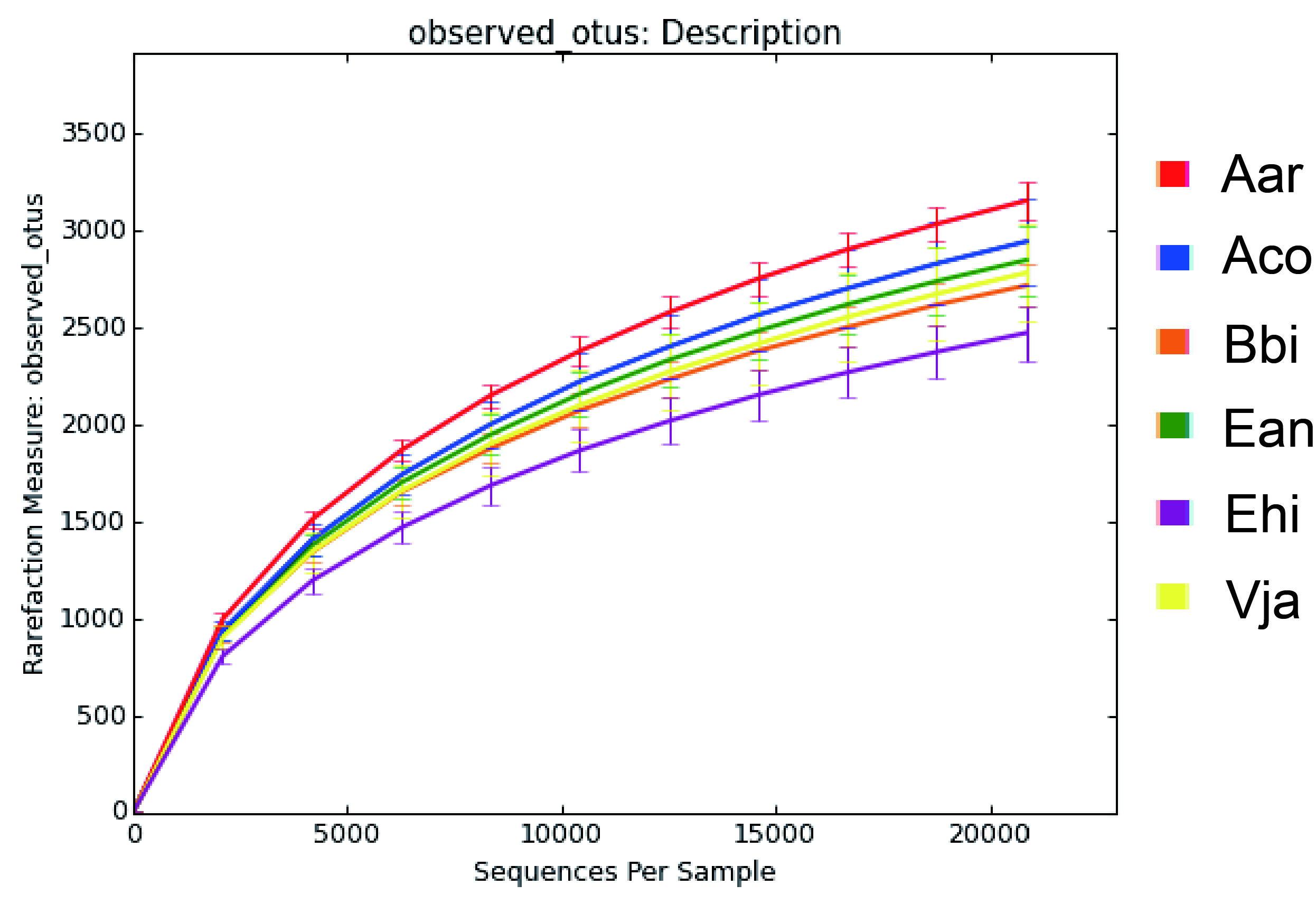


**Fig. S3** **The OTU number boxplot (A) and the rank abundance curve (B) to indicate the bacterial alpha-diversity and richness in six plant rhizosphere soils.** The plots were drawn using the average value of five replicates of each sample group. Aco, *Ageratum conyzoides*; Ean, *Erigeron annuus*; Bbi, *Bidens biternata*; Aar, *Artemisia argyi*; Vja, *Viola japonica*; Ehi, *Euphorbia hirta*.


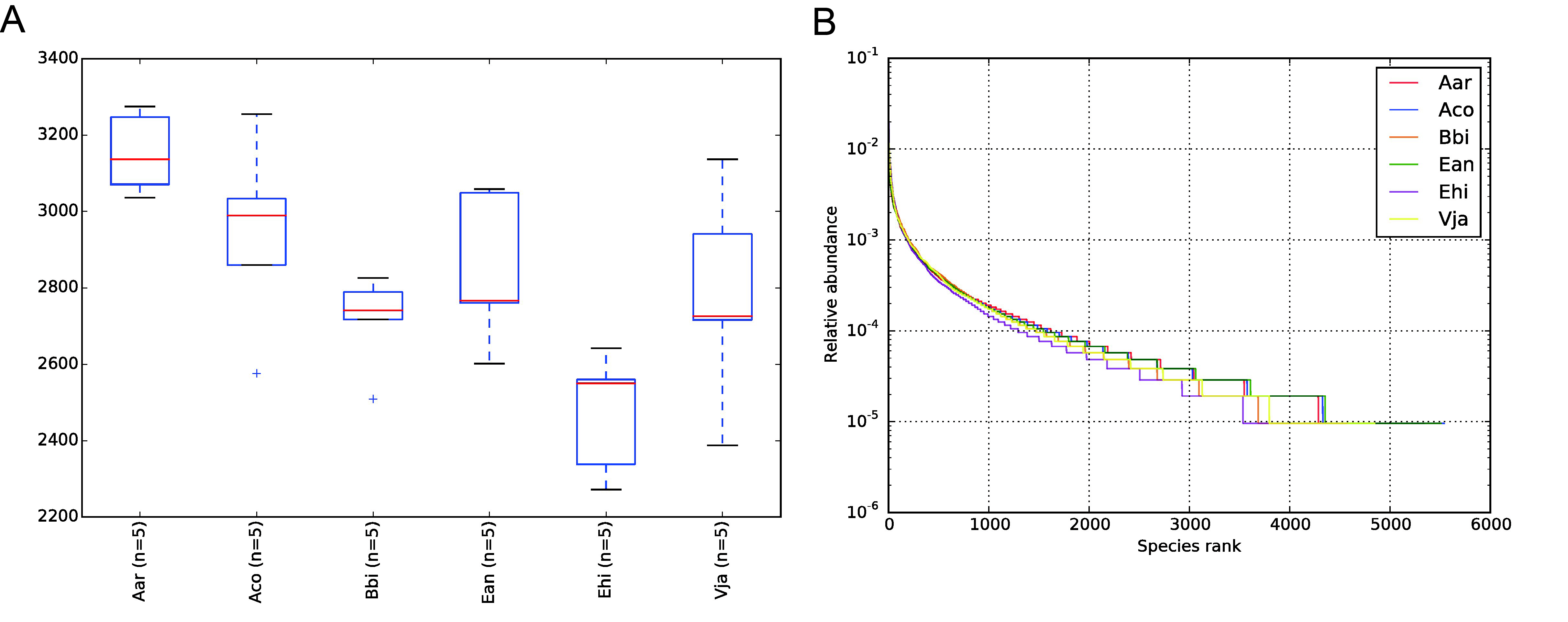


**Fig. S4** **Canonical Analysis of Principle Coordinates (CAP) was computed using the function capscale() from the R Package Vegan**. Aco, *Ageratum conyzoides*; Ean, *Erigeron annuus*; Bbi, *Bidens biternata*; Aar, *Artemisia argyi*; Vja, *Viola japonica*; Ehi, *Euphorbia hirta*.


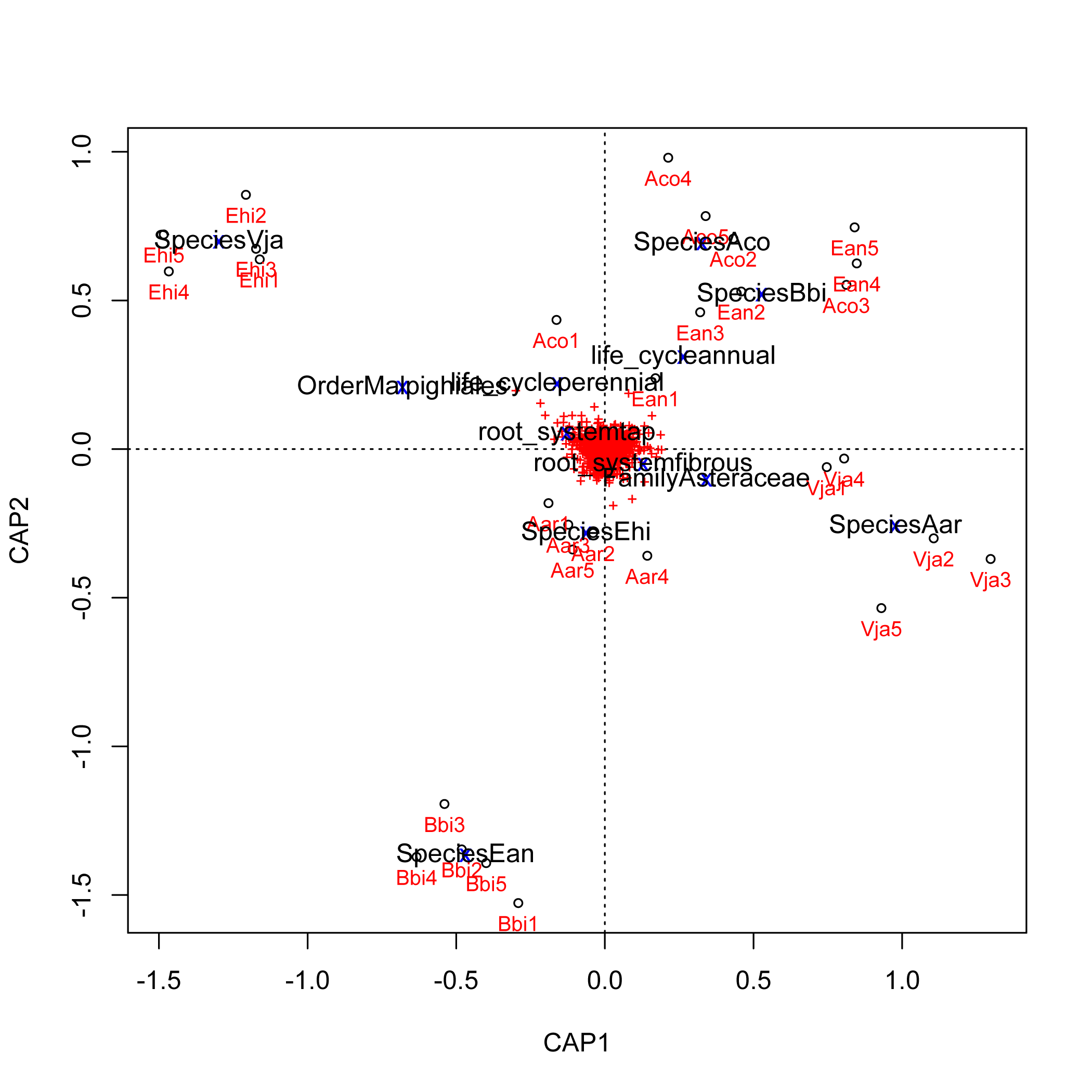

Supplement: Supplementary file 1 [file MBO3-8-e00762-s001.doc]
